# Diphtheria

**Published:** 1864-11

**Authors:** 


For sale by W. B. Keen & Co., 148 Lake Street, Chicago.

Diphtheria. Its Nature and Treatment. With an account of the History of its Prevalence in various Countries. By Daniel Slade, M.D. Being a second and revised edition of an Essay, to which was awarded the Fisk Fund Prize for 1860. Philadelphia: Blanchard & Lea. 1864.

This is a neatly published volume of 166 pages. And of the many monographs written on the subject of diphtheria, during the last five or six years, this is one of the best.

